# The SIRS criteria have better performance for predicting infection than qSOFA scores in the emergency department

**DOI:** 10.1038/s41598-020-64314-8

**Published:** 2020-05-15

**Authors:** Satoshi Gando, Atsushi Shiraishi, Toshikazu Abe, Shigeki Kushimoto, Toshihiko Mayumi, Seitaro Fujishima, Akiyoshi Hagiwara, Yasukazu Shiino, Shin-ichiro Shiraishi, Toru Hifumi, Yasuhiro Otomo, Kohji Okamoto, Junichi Sasaki, Kiyotsugu Takuma, Kazuma Yamakawa, The Japanese Association for Acute Medicine (JAAM) Sepsis Prognostication in Intensive Care Unit and Emergency Room (SPICE) (JAAM SPICE) Study Group, Atsumi Hoshino, Atsushi Shiraishi, Toshiaki Abe, Manabu Sugita, Yoshihiro Hanaki, Akiyoshi Hagiwara, Shin-ichiro Shiraishi, Yasukazu Shiino, Masahiro Harada, Hideaki Yoshihara, Kiyotsugu Takuma, Yasuhiro Otomo, Kazuma Morino, Yoshihiro Shimizu, Hiroyasu Ishikura, Toru Hifumi, Yoshizumi Deguchi, Sho Nachi, Satoshi Gando, Kohji Okamoto, Masato Kawakami, Seitaro Fujishima, Junichi Sasaki, Junichi Maehara, Kunihiko Okada, Kazuma Yamakawa, Kazuya Kiyota, Yasuo Miki, Kaoru Koike, Takashi Muroya, Hisashi Yamashita, Toshihiko Mayumi, Hideaki Anan, Tadashi Kaneko, Hirotada Kittaka, Hiroyuki Yamaguchi

**Affiliations:** 1 Division of Acute and Critical Care Medicine, Hokkaido University Graduate School of Medicine, Sapporo, Japan; 2 Department of Acute and Critical Care Medicine, Sapporo Higashi Tokushukai Hospital, Sapporo, Japan; 3 Emergency and Trauma Center, Kameda Medical Center, Kameda, Japan; 4 Department of General Medicine, Juntendo University, Tokyo Japan; Health Services Research and Development Center, University of Tsukuba, Tsukuba, Japan; 5 Division of Emergency and Critical Care Medicine, Tohoku University Graduate School of Medicine, Sendai, Japan; 6 Department of Emergency Medicine, School of Medicine, University of Occupational and Environmental Health, Fukuoka, Japan; 7 Center for General Medicine Education, Keio University School of Medicine, Tokyo, Japan; 8 Center Hospital of the National Center for Global Health and Medicine, Tokyo Japan; Department of Emergency Medicine, Niizashiki Chuo General Hospital, Tokyo, Japan; 9 Department of Acute Medicine, Kawasaki Medical School, Kawasaki, Japan; 10 Department of Emergency and Critical Care Medicine, Aizu Chuo Hospital, Aizu, Japan; 11 Department of Emergency and Critical Care Medicine, St. Luke’s International Hospital, Tokyo, Japan; 12 Trauma and Acute Critical Care Center, Medical Hospital, Tokyo Medical and Dental University, Tokyo, Japan; 13 Department of Surgery, Center for Gastroenterology and Liver Disease, Kitakyushu City Yahata Hospital, Yahata, Japan; 14 Department of Emergency and Critical Care Medicine, Keio University School of Medicine, Tokyo, Japan; 15 Emergency & Critical Care Center, Kawasaki Municipal Kawasaki Hospital, Kawasaki, Japan; 16 Division of Trauma and Surgical Care, Osaka General Medical Center, Osaka, Japan; 17 Public Toyooka Hospital,Tajima Emergency & Critical Care Medical Center, Hyogo, Japan; 18 Kameda Medical Center, Kamogawa, Japan; 19 Juntendo University Urayasu Hospital, Urayasu, Japan; 20 Juntendo University Nerima Hospital, Urayasu, Japan; 21 Japanese Red Cross Nagoya Daiichi Hospital, Nagoya, Japan; 22 Center Hospital of the National Center for Global Health and Medicine, Tokyo, Japan; 23 Aizu Chuo Hospital, Aizuwakamatsu, Japan; 24 Kawasaki Medical School Hospital, Kurashiki, Japan; 25 National Hospital Organization Kumamoto Medical Center, Kumamoto, Japan; 26 Kagoshima City Hospital, Kagoshima, Japan; 27 Kawasaki Municipal Kawasaki Hospital, Kawasaki, Japan; 28 Tokyo Medical and Dental University Hospital, Tokyo, Japan; 29 Yamagata Prefectural Central Hospital, Yamagata, Japan; 30 Kyoto Okamoto Memorial Hospital, Kyoto, Japan; 31 Fukuoka University Hospital, Fukuoka, Japan; 32 Kagawa University Hospital, Kagawa, Japan; 33 Tokyo Women’s Medical University Medical Center East, Tokyo, Japan; 34 Gifu University Hospital, Gifu, Japan; 35 Hokkaido University Graduate School of Medicine, Hokkaido, Japan; 36 Kitakyushu City Yahata Hospital, Kitakyushu, Japan; 37 Ome Municipal General Hospital, Ome, Japan; 38 Keio University Hospital, Keio, Japan; 39 Saiseikai Kumamoto Hospital, Kumamoto, Japan; 40 Saku Central Hospital, Saku, Japan; 41 Osaka General Medical Center, Osaka, Japan; 42 Saitama Red Cross Hospital, Saitama, Japan; 43 Fujieda Municipal General Hospital, Fujieda, Japan; 44 Kyoto University Hospital, Kyoto, Japan; 45 Kansai Medical University Hospital, Kansai, Japan; 46 St. Mary’s Hospital, Kurume, Japan; 47 University of Occupational and Environmental Health Hospital, Kitakyushu, Japan; 48 Fujisawa City Hospital, Fujisawa, Japan; 49 Kumamoto University Hospital, Kumamoto, Japan; 50 Osaka Mishima Emergency Critical Care Center, Osaka, Japan; 51 Seirei Yokohama Hospital, Yokohama, Japan

**Keywords:** Infectious diseases, Prognosis

## Abstract

Systemic inflammatory response syndrome (SIRS) reportedly has a low performance for distinguishing infection from non-infection. We explored the distribution of the patients diagnosed by SIRS (SIRS patients) or a quick sequential organ failure assessment (qSOFA) (qSOFA patients) and confirmed the performance of the both for predicting ultimate infection after hospital admission. We retrospectively analyzed the data from a multicenter prospective study. When emergency physicians suspected infection, SIRS or the qSOFA were applied. The area under the receiver operating characteristic curves (AUC) was used to assess the performance of the SIRS and qSOFA for predicting established infection. A total of 1,045 patients were eligible for this study. The SIRS patients accounted for 91.6% of qSOFA patients and they showed a higher rate of final infection than that of non-SIRS patients irrespective of the qSOFA diagnosis. The AUCs for predicting infection with SIRS and a qSOFA were 0.647 and 0.582, respectively. The SIRS significantly predicted an ultimate infection (AUC, 0.675; p = 0.018) in patients who met the SIRS and qSOFA simultaneously. In conclusion, the SIRS patients included almost all qSOFA patients. SIRS showed a better performance for predicting infection for qSOFA in those who met both definitions.

## Introduction

Since the announcement of the third international consensus definitions for sepsis and septic shock (Sepsis-3), much debate has been had on the accuracy of the quick sequential organ failure assessment (qSOFA) score for predicting mortality due to sepsis compared with the systemic inflammatory response syndrome (SIRS) criteria^1–3^. Systematic reviews and meta-analyses in various settings, e.g. emergency departments, intensive-care units (ICUs), and general wards have consistently demonstrated a high sensitivity and low specificity with SIRS criteria but a low sensitivity and high specificity with the qSOFA score for predicting hospital mortality in patients suspected of or with an infection^4–10^. Herwanto *et al*.^11^ robustly confirmed these results presenting the area under the receiver operating characteristic (ROC) curve (AUC) and noted that neither score is perfect, each having its own limitations.

One reasons the Sepsis-3 criteria were proposed was under the definition of SIRS, systemic inflammation due to infectious and non-infectious insults such as pancreatitis and trauma, it is difficult to differentiate sepsis from noninfectious insults^1,3^. No difference in the accuracy for diagnosing sepsis defined by the Sepsis-3 criteria has been reported between SIRS criteria and qSOFA scores^10^; however, another systematic review and meta-analysis reported the significantly superior performance of the SIRS criteria to the qSOFA score for diagnosing sepsis in patients outside the ICU^12^. One unresolved issue is the performance of the SIRS criteria and qSOFA score for predicting established infection as distinguished from noninfectious insults in patients with suspected infection. A previous report found that infectious patients who met the SIRS criteria included almost all of those who met qSOFA definition in the emergency department^13^. Another issue to be resolved is the degree of overlap between the patients who met the SIRS criteria (SIRS patients) and those who met qSOFA definition (qSOFA patients) in patients with suspected infection.

The objectives of this study conducted in an emergency department were exploring the distribution of the patients with suspected infection diagnosed based on the SIRS criteria or qSOFA score and to confirm the performance of the SIRS criteria and qSOFA score for predicting an ultimate infection diagnosis after hospital admission as distinguished from noninfectious insults.

## Methods

### Study design, setting, and ethical approval

This is a retrospective prognostic study used the data of the Emergency Room (ER) cohort from the prospective, multicenter study of the Japanese Association for Acute Medicine (JAAM) Sepsis Prognostication in Intensive Care Unit and Emergency Room (SPICE) study, comprising the SPICE-ER and SPICE-ICU cohort. The main study of the SPICE-ER cohort externally validated the accuracy of the SIRS criteria and qSOFA score for predicting mortality in patients suspected of having an infection in the emergency department. The JAAM SPICE-ER study used samples from 35 emergency departments in tertiary hospitals. The patient recruitment and data collection were conducted from December 2017 to February 2018 and the included patients were followed up to their discharge of the hospital. The JAAM SPICE-ER was registered in the University Hospital Medical Information Network Clinical Trial Registry (UMIN-CTR ID: UMIN000027258).

This study was conducted in accordance with the amended Declaration of Helsinki, and was approved by the JAAM and the Ethics Committee of each hospital waiving written informed consent (JAAM, 2016-01; Hokkaido University Graduate School of Medicine, head institute of the SPICE group, 016-0385).

### Participants

The JAAM SPICE-ER study enrolled patients >16 years old who (1) were suspected of having an infection by the emergency physicians and (2) had received any kind of antibiotics, had their body fluid cultured, or had imagining done for the detection of infection sites during their stay in the emergency department. Patients were excluded if they were transferred to another hospital without first being hospitalized at the participating hospital.

### Variables, definition, and outcome measures

In addition to the baseline characteristics of the patients, the clinical frailty index^14^, Charlson comorbidity index^15^, lactate levels, and parameters for calculating SIRS criteria and qSOFA score were obtained. The SIRS criteria were defined according to the original consensus study (Sepsis-1)^2^ and the qSOFA score was based on the Sepsis-3 definition^1^. SIRS criteria >2 and qSOFA score >2 met the definition of SIRS and qSOFA, respectively. The suspected infection sites were classified into 12 regions, including the respiratory tract, urinary tract, abdomen, central nervous system, skin and soft tissue, bone and joint, wounds, intravascular catheter, endocardium, any kind of implant aside from an intravascular catheter, others, and unknown origin. The ultimately confirmed sites of infection after hospitalization were classified into the same categories and final diagnosis of infection or non-infection was also determined after admission to the hospital. The primary outcome of this study was an ultimate diagnosis of infection after admission.

### Statistical analyses

The statistical parameters required for the original study sample size estimation were not available from previous studies, therefore, the original study employed an adaptive sample size estimation design.

Numeric variables are expressed as the median with the 25^th^–75^th^ interquartile range and nominal variables are shown as the number (percentage). The chi-square test or Fisher’s exact test for nominal variables was used when required. The ROC curve was constructed, and the AUC was used to assess the predictive ability of an ultimate infection diagnosis. Missing values were used without manipulation. Differences with a two-tailed p value of <0.05 were considered statistically significant. The IBM SPSS 25.0 for MAC OSX software program (IBM Japan, Tokyo, Japan) was used for the statistical analyses and calculations.

## Results

### Demographics and characteristics of the patients

**Figure 1:**
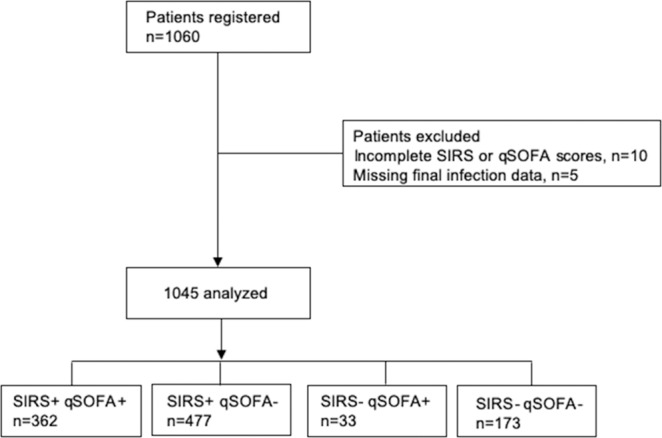
Flow diagram of the study. qSOFA, quick sequential organ failure assessment; SIRS, systemic inflammatory response syndrome.

**Figure 2:**
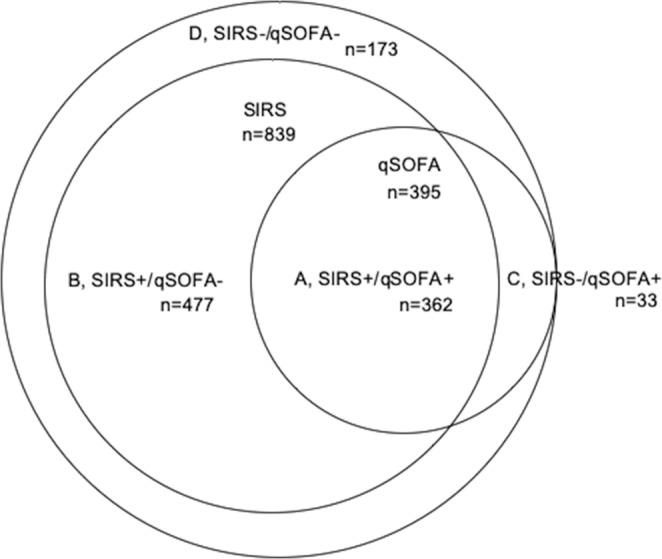
Distribution of the patients with suspected infection who presented to the emergency department. SIRS refers to patients who met >2 SIRS criteria, and qSOFA refers to patients with qSOFA score >2. (**A**), patients met both the SIRS criteria and qSOFA score; (**B**), patients met only the SIRS criteria; (**C**), patients met only the qSOFA score; (**D**), patients met neither the SIRS criteria nor qSOFA score. The SIRS patients included almost all (91.6%) qSOFA patients. qSOFA, quick sequential organ failure assessment; SIRS, systemic inflammatory response syndrome.

The flow diagram showing the study population as well as inclusions and exclusions is presented in Fig. 1. Of the 1,060 registered patients, a total of 1,045 patients were ultimately analyzed. Table 1 shows the characteristics of the patients who met the SIRS criteria or qSOFA definition. As shown in Fig. 2, there were huge overlaps between the two groups.

**Table 1:** Baseline characteristics of the patients.

	SIRS(n = 839)	qSOFA(n = 395)
Age (years)	78 (65–84)	81 (71–86)
Male n (%)	502 (59.8)	232 (58.7)
Charlson comorbidity index	2 (2–4)	2 (2–3)
Clinical frailty index	4 (3–6)	5 (3–7)
SIRS criteria	3 (2–3)	3 (2–3)
qSOFA score	1 (1–2)	2 (2–3)
Respiratory rate/min	24 (20–29)	26 (23–30)
PCO_2_ (mmHg)	36.2 (30.1–43.4)	35.0 (29.1–44.0)
Heart rate/min	102 (90–117)	102886–119)102(86-119)
Temperature (Celsius)	37.8 (36.7–38.8)	37.4 (36.5–38.7)
White blood cell counts/mm^3^	11,500 (7,550–15,630)	10,800 (6,850–15,200)
Systolic blood pressure (mmHg)	125 (102–147)	102 (87–136)
Glasgow Coma Scale	14 (13–15)	13 (10–14)
Lactate (mmol/L)	1.9 (1.3–3.5)	2.6 (1.5–4.7)
**Final sites of infection n (%)**		
Respiratory system	394 (47.0)	208 (52.7)
Abdomen	147 (17.5)	53 (13.4)
Central nervous system	2 (0.2)	3 (0.8)
Skin and soft tissue	35 (4.2)	11 (2.8)
Bone and joint	5 (0.6)	2 (0.5)
Wounds	2 (0.2)	0 (0)
Catheter	2 (0.2)	3 (0.8)
Endocardium	6 (0.7)	2 (0.5)
Implant	2 (0.2)	0 (0)
Other	34 (4.1)	14 (3.5)
Unknown	31 (3.7)	15 (3.8)
No infection	54 (6.4)	19 (4.8)
Mortality n(%)	128 (15.3)	84 (21.3)

SIRS, systemic inflammatory response syndrome; qSOFA, quick sequential organ failure assessment. All data were obtained at the time infection was suspected in the emergency department. Numeric variables are presented as the median and 25^th^ to 75^th^ percentile and nominal variables as the number and percentage.

### Distribution of the patients diagnosed by the SIRS criteria and qSOFA score

Figure 2 shows the incidence of SIRS criteria- and qSOFA-based diagnosis, indicating the overlap between SIRS patients and qSOFA patients. Almost all patients (80.3%, 839/1045) met the SIRS criteria, while 395 (37.8%) patients met the qSOFA definition. The SIRS patients included 91.6% (362/395) of the patients who met the qSOFA definition. One hundred and seventy-three patients met neither the SIRS criteria nor the qSOFA definition.

### Prediction of a final diagnosis of infection

**Figure 3:**
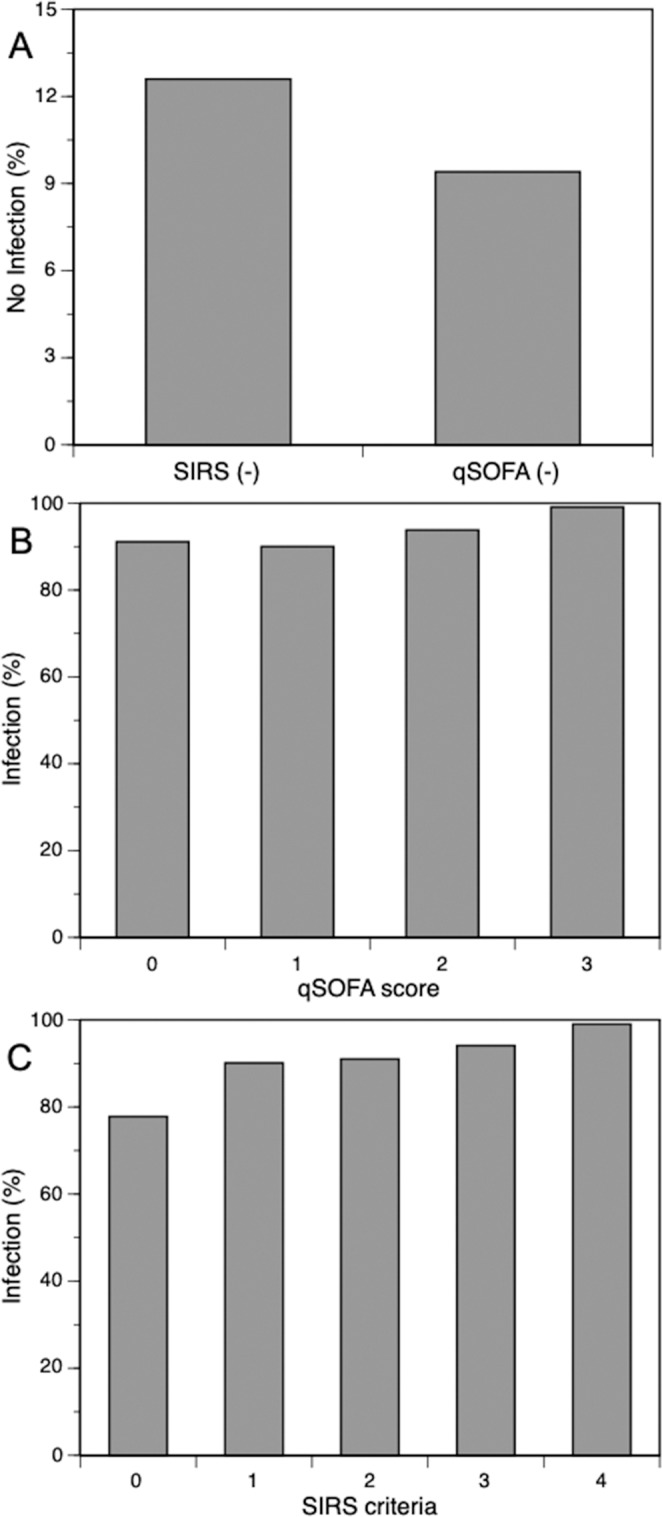
Bar graphs showing the prevalence of an ultimate infection after admission. Both SIRS (p < 0.001) and qSOFA (p = 0.015) showed stepwise increases in the rates of infection in parallel with the increases in the number of criteria and scores, respectively. Rates of patients without infection among non-SIRS patients (12.6%) tended to higher than among non-qSOFA patients (9.4%). SIRS (−), non-SIRS patients who did not meet SIRS criteria >2; qSOFA (−), non-qSOFA patients who did not meet qSOFA >2. qSOFA, quick sequential organ failure assessment; SIRS, systemic inflammatory response syndrome.

Table 2 shows significant differences in the percentage of final diagnosis of infection among 4 groups (p = 0.04). The patients who simultaneously met both the SIRS criteria and qSOFA definition showed a higher prevalence of an ultimate diagnosis of infection (95.6%) than the other groups and the highest mortality rate (20.4%). The SIRS patients had a higher percentage of an ultimate diagnosis of infection than the non-SIRS patients irrespective meeting the qSOFA definition (785/839, 93.6% vs. 180/206, 87.4%, p = 0.005). Both SIRS (p < 0.001) and qSOFA (p = 0.015) showed stepwise increases in the rates of infection in parallel with the increases in the number of criteria and scores, respectively. Of note however, the rates of non-infectious patients among non-SIRS patients (12.6%) tended to higher than among non-qSOFA patients (9.4%). The results are shown in Fig. 3.

**Table 2:** Rates of hospital mortality and positive infection as the final diagnosis.

	A	B	C	D
	SIRS +/qSOFA+	SIRS +/qSOFA−	SIRS −/qSOFA+	SIRS −/qSOFA-
	n = 362	n = 477	n = 33	n = 173
Mortality n (%)*	74 (20.4)	54 (11.3)	10 (30.3)	15 (8.7)
Infection n (%)**	346 (95.6)	439 (92.0)	30 (90.9)	150 (86.7)

A, B, C and D are same as those in Fig. 2. *P < 0.001, **p = 0.004.

**Figure 4:**
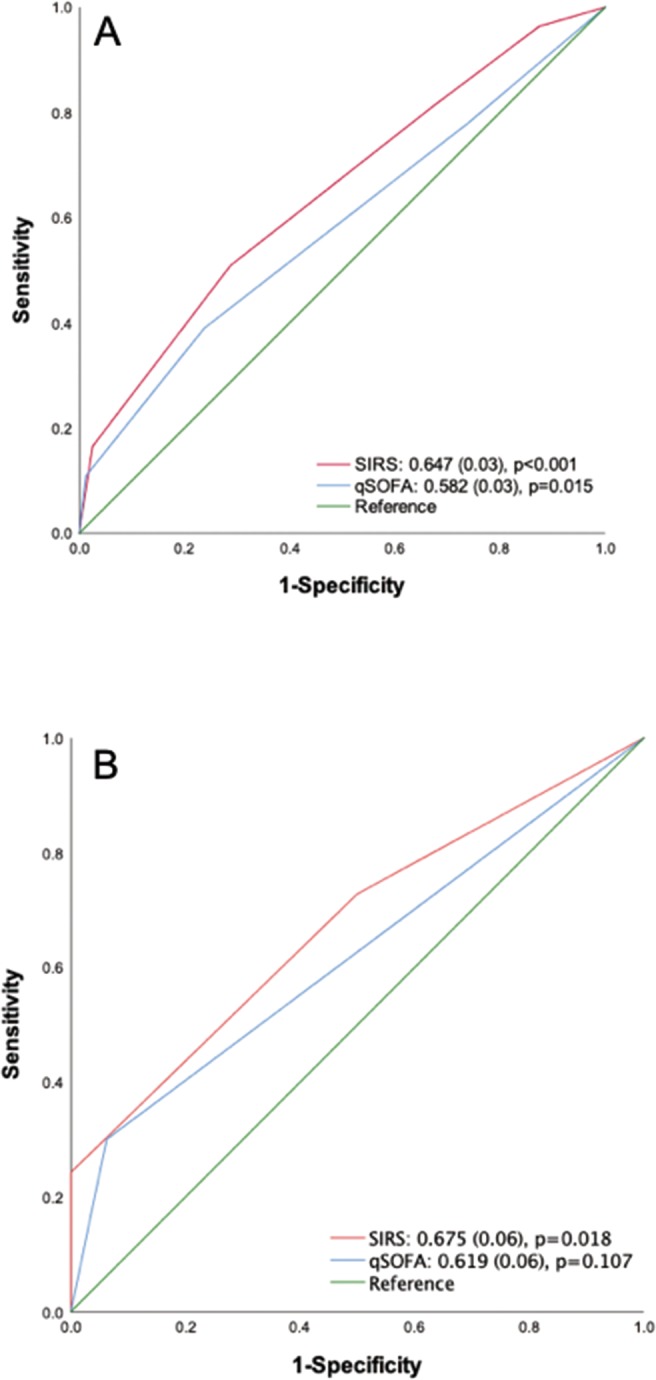
Receiver operating characteristic (ROC) curve analyses for predicting an ultimately established diagnosis of infection in patients with suspected infection at the presentation to the emergency department. (**A**), All patients presented to the emergency department; (**B**), Patients who met both the SIRS criteria and qSOFA score. Numbers indicate the AUC (SE), p-value. AUC, area under the ROC curve; qSOFA, quick sequential organ failure assessment; SE, standard error; SIRS, systemic inflammatory response syndrome.

Figure 4A shows that the AUC of the SIRS criteria was significant for predicting an ultimate infection diagnosis (AUC, 0.647; standard error [SE], 0.03, p < 0.001), with a sensitivity of 81.3% and specificity of 32.5%. The AUC of the qSOFA score for predicting an established infection diagnosis was 0.582 (SE 0.03) (p = 0.015), which was narrower than that of SIRS criteria. In patients with suspected of having an infection who both met SIRS criteria and fit the qSOFA definition simultaneously, the SIRS criteria were significantly more accurate for predicting an ultimate infection diagnosis than the qSOFA score (AUC, 0.675; SE, 0.06, p = 0.018). The AUC of qSOFA non-significantly predicted an ultimate infection diagnosis (AUC, 0.619; SE0.06, p = 0.107) (Fig. 4B).

## Discussion

### Brief summary

According to the present study, the SIRS patients included almost all qSOFA patients, and the SIRS patients showed a higher percentage of an ultimately established diagnosis of infection than the non-SIRS patients irrespective of the qSOFA score. The SIRS criteria, especially in patients who met both the SIRS criteria and the qSOFA score, showed a significant AUC for predicting an ultimate infection diagnosis after admission to the hospital.

A systematic review and meta-analysis including a large patient population showed that the qSOFA score had greater ability than the SIRS criteria for predicting sepsis mortality and secondary outcomes such as organ dysfunction, ICU admission, ventilatory support, a prolonged ICU stay, and the 30-day outcome^11^. However, information regarding an established diagnosis of infection, sepsis, and septic shock was lacking in that meta-analysis. The main reason for replacing the sepsis definition in 1992 (Sepsis-1)^2^ with Sepsis-3^3^ was that SIRS criteria were extremely sensitive, leading to the misdiagnosis of non-infectious insults such as trauma, burns, pancreatitis, and ischemia-reperfusion events, as true infection^16^. However, in contrast to those previous findings, the present study showed a good predictive ability of the SIRS criteria for an ultimate diagnosis of infection.

In the current study, the AUC of the qSOFA score for predicting real infection was narrower than that of the SIRS criteria in patients with suspected infection. In addition, a non-significant AUC of the qSOFA score for predicting established infection was observed in patients who met both the SIRS criteria and qSOFA definitions. Not all patients with infection progress to sepsis. However, a systematic review and meta-analysis concluded that the SIRS criteria were significantly more accurate than the qSOFA score for diagnosing sepsis according to Sepsis-3^12^. The results of present study and this meta-analysis are inevitable as the qSOFA score has been established and validated as a prognostic tool for hospital death in the patients with suspected infection^17^, while the SIRS criteria are used as a screening tool for severe sepsis which is defined as systemic inflammation with organ dysfunction according to Sepsis-1^2^.

Despite the above issues, the international consensus of Sepsis-3 used the qSOFA score as a screening tool for diagnosing sepsis, namely dysregulated host responses to infection associated with organ dysfunction (SOFA > 2)^1^. In the first large validation study of Sepsis-3 in patients suspected of having infection who presented to the emergency department, the patients without infection were excluded from the validation, and the conclusion was that the qSOFA score had a greater prognostic accuracy for hospital mortality than the SIRS criteria^18^. There may be some inconsistencies between the original paper and the validation study^1,18^. It is a time to become aware that the primary outcome of the study attempting to compare the SIRS criteria and qSOFA score is not the prediction of hospital mortality but the prediction of infection that progresses to sepsis or prediction of sepsis itself.

Among patients with suspected infection, a significant portion (91.6%) of those who met the qSOFA definition were included in those who met the SIRS criteria. These results were consistent with those obtained by Henning *et al*.^13^, who showed that the SIRS patients include almost all qSOFA patients among the infectious patients. The present and previous findings suggest that the SIRS criteria can replace the qSOFA score as a screening tool for sepsis in patients with suspected infection^1^. Alternatively, the combined application of those two tests for patients with suspected infection may improve the accuracy of both as screening and prognostic tools.

On comparing the SIRS criteria, qSOFA score, and the National Early Warning score (NEWS), the qSOFA score had the lowest sensitivity and was recognized as a poor tool for use in emergency department sepsis screening^19^. That study further showed that the NEWS was more accurate for detecting sepsis than the SIRS criteria (AUC of NEWS vs. SIRS criteria: 0.91 vs. 0.88) and recommended the NEWS as a screening tool for sepsis in the emergency department. However, screening of sepsis should be performed based on the pathophysiology of sepsis rather than using the simple warning score like the NEWS. Namely, SIRS, defined as systemic inflammatory responses to the infection would be good screening tool for sepsis, because the SIRS criteria are based on the sepsis pathophysiology as described in Sepsis-1^2^.

### Limitations

The strength of this study was our use of prospective data collected by the emergency physicians immediately after presentation to the emergency department. However, several limitations associated with the present study also warrant mention. The retrospective nature of the analyses may have limited the robustness of the study. The sample size was determined for the validation of hospital mortality predicted by the SIRS criteria and qSOFA score in the original study. Diagnostic data on non-infectious insults were lacking. The present study was a single national study conducted in a developed country in an emergency department setting, which may limit the generalizability of the obtained results. However, we believe that our study highlighted important discussion points for the future studies, supporting the further comparison of the SIRS criteria and qSOFA score.

## Conclusions

In the emergency department patients with suspected infection, the SIRS patients included almost all qSOFA patients and were associated with higher incidence of an ultimate infection diagnosis than non-SIRS patients irrespective of the qSOFA diagnosis. The SIRS criteria were significantly more accurate in predicting an established infection, especially in those who met both the SIRS criteria and the qSOFA definition. These results may suggest that the qSOFA score can be replaced with the SIRS criteria as a screening tool of infection likely to progress to sepsis. Alternatively, the combined application of both the SIRS criteria and qSOFA score in patients with suspected of having infection may improve the screening and prognostic accuracy of these factors for predicting infection and/or sepsis and a poor outcome.

### Ethical approval and consent to participate

This study was approved by the JAAM and the Ethics Committee of each hospital waiving written informed consent (JAAM, 2016-01; Hokkaido University Graduate School of Medicine, head institute of the SPICE group, 016-0385).

## Data Availability

The dataset used and/or analyzed during current study are available from the corresponding author on reasonable request.
